# 5-Chloro-2-methyl-3-(4-methyl­phenyl­sulfon­yl)-1-benzo­furan

**DOI:** 10.1107/S1600536813015468

**Published:** 2013-06-08

**Authors:** Hong Dae Choi, Pil Ja Seo, Uk Lee

**Affiliations:** a Department of Chemistry, Dongeui University, San 24 Kaya-dong, Busanjin-gu, Busan 614-714, Republic of Korea; b Department of Chemistry, Pukyong National University, 599-1 Daeyeon 3-dong, Nam-gu, Busan 608-737, Republic of Korea

## Abstract

The title compound, C_16_H_13_ClO_3_S, crystallized with two independent mol­ecules in the asymmetric unit. The 4-methyl­phenyl rings make dihedral angles of 75.15 (4)° and 72.18 (4)° with the planes of the benzo­furan ring systems in the two mol­ecules. In the crystal, mol­ecules are linked by weak C—H⋯O and C—H⋯π inter­actions, forming a three-dimensional network.

## Related literature

For background information and the crystal structures of related compounds, see: Choi *et al.* (2010 ▶, 2012 ▶).
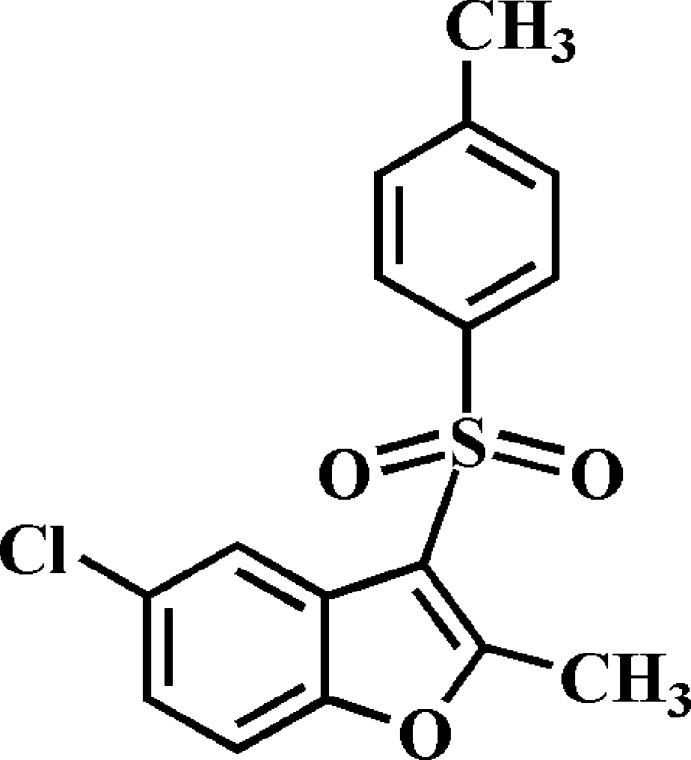


## Experimental

### 

#### Crystal data

C_16_H_13_ClO_3_S
*M* *_r_* = 320.77
Triclinic, 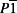

*a* = 7.3725 (2) Å
*b* = 10.0967 (3) Å
*c* = 20.8173 (7) Å
α = 98.086 (1)°
β = 99.547 (2)°
γ = 106.547 (1)°
*V* = 1435.62 (8) Å^3^
*Z* = 4
Mo *K*α radiation
μ = 0.42 mm^−1^
*T* = 173 K
0.41 × 0.29 × 0.23 mm

#### Data collection

Bruker SMART APEXII CCD diffractometer
Absorption correction: multi-scan (*SADABS*; Bruker, 2009 ▶) *T*_min_ = 0.670, *T*_max_ = 0.746
26328 measured reflections
7109 independent reflections
5967 reflections with *I* > 2σ(*I*)
*R*_int_ = 0.028

#### Refinement

*R*[*F* ^2^ > 2σ(*F* ^2^)] = 0.036
*wR*(*F* ^2^) = 0.098
*S* = 1.04
7109 reflections
383 parameters
H-atom parameters constrained
Δρ_max_ = 0.38 e Å^−3^
Δρ_min_ = −0.40 e Å^−3^

### 

Data collection: *APEX2* (Bruker, 2009 ▶); cell refinement: *SAINT* (Bruker, 2009 ▶); data reduction: *SAINT*; program(s) used to solve structure: *SHELXS97* (Sheldrick, 2008 ▶); program(s) used to refine structure: *SHELXL97* (Sheldrick, 2008 ▶); molecular graphics: *ORTEP-3 for Windows* (Farrugia, 2012 ▶) and *DIAMOND* (Brandenburg, 1998 ▶); software used to prepare material for publication: *SHELXL97*.

## Supplementary Material

Crystal structure: contains datablock(s) I. DOI: 10.1107/S1600536813015468/pk2485sup1.cif

Structure factors: contains datablock(s) I. DOI: 10.1107/S1600536813015468/pk2485Isup2.hkl

Supplementary material file. DOI: 10.1107/S1600536813015468/pk2485Isup3.cml

Additional supplementary materials:  crystallographic information; 3D view; checkCIF report

## Figures and Tables

**Table 1:** Hydrogen-bond geometry (Å, °) *Cg* is the centroid of the C26–C31 ring.

*D*—H⋯*A*	*D*—H	H⋯*A*	*D*⋯*A*	*D*—H⋯*A*
C15—H15⋯O2^i^	0.95	2.56	3.251 (2)	130
C27—H27⋯O6^ii^	0.95	2.55	3.248 (2)	131
C30—H30⋯O3^iii^	0.95	2.48	3.361 (2)	154
C22—H22⋯*Cg* ^iv^	0.95	2.71	3.572 (2)	136

Symmetry codes: (i) 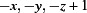; (ii) 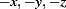; (iii) 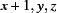; (iv) 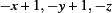.

**Fig. 1.**
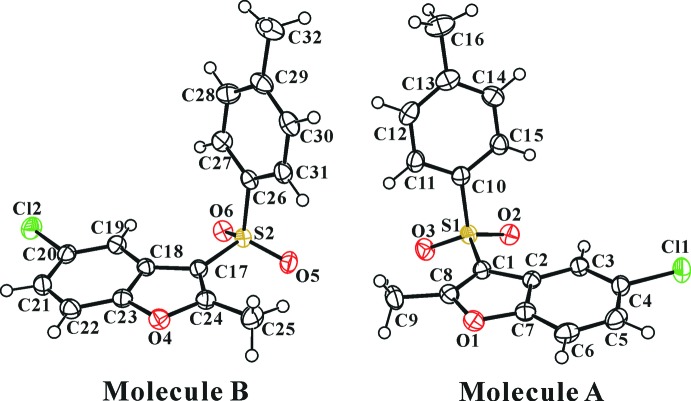
The molecular structure of the title compound with the atom numbering scheme. Displacement ellipsoids are drawn at the 50% probability level.

**Fig. 2.**
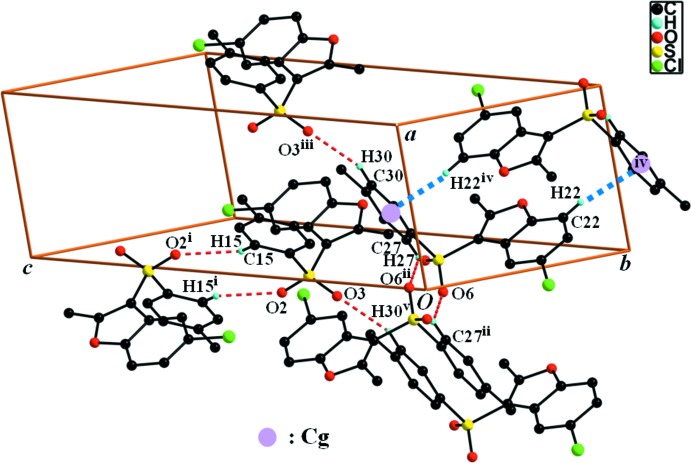
A view of the C—H···O and C—H···π interactions (dotted lines) in the crystal structure of the title compound. H atoms non-participating in hydrogen-bonding were omitted for clarity. [Symmetry codes: (i) - x, - y, - z + 1; (ii) - x, - y, - z; (iii) x + 1, y, z; (iv) - x + 1, - y + 1, - z; (v) - x, - y, - z.]

## supplementary crystallographic information

### Comment

As a part of our continuing study of 5-chloro-2-methyl-1-benzofuran derivatives containing 4-fluorophenylsulfonyl (Choi *et al.*, 2010) and 4-bromophenylsulfonyl (Choi *et al.*, 2012) substituents in 3-postion, we report herein the crystal structure of the title compound which crystallizes with two symmetrically independent molecules, A & B, in the asymmetric unit.

In the title molecule (Fig. 1), the benzofuran unit is essentially planar, with a mean deviation of 0.006 (1) and 0.007 (1) Å, for A and B, respectively, from the least-squares plane defined by the nine constituent atoms. The dihedral angles between the 4-methylphenyl ring and the mean plane of the benzofuran ring system are 75.15 (4)° in molecule A and 72.18 (4)° in molecule B, respectively. In the crystal packing (Fig. 2), molecules are connected by weak C—H···O and C—H···π interactions (Table 1, Cg is the centroid of the C26–C31 4-methylphenyl ring), forming a three-dimensional network.

### Experimental

3-Chloroperoxybenzoic acid (77%, 560 mg, 2.5 mmol) was added in small portions to a stirred solution of 5-chloro-2-methyl-3-(4-methylphenylsulfanyl)-1-benzofuran (346 mg, 1.2 mmol) in dichloromethane (40 mL) at 273 K. After being stirred at room temperature for 10h, the mixture was washed with saturated sodium bicarbonate solution and the organic layer was separated, dried over magnesium sulfate, filtered and concentrated at reduced pressure. The residue was purified by column chromatography (hexane–ethyl acetate, 4:1 v/v) to afford the title compound as a colorless solid [yield 68%, m.p. 461–462 K; *R*_f_ = 0.64 (hexane-ethyl acetate, 4:1 v/v)]. Single crystals suitable for X-ray diffraction were prepared by slow evaporation of a solution of the title compound in benzene at room temperature.

### Refinement

All H atoms were positioned geometrically and refined using a riding model, with C—H = 0.95 Å for aryl and 0.98Å for methyl H atoms. *U*_iso_(H) = 1.2*U*_eq_(C) for aryl and 1.5*U*_eq_(C) for methyl H atoms. The positions of methyl hydrogens were optimized rotationally.

### Crystal data

**Table:**

C_16_H_13_ClO_3_S	*Z* = 4
*M**_r_* = 320.77	*F*(000) = 664
Triclinic, *P*1	*D*_x_ = 1.484 Mg m^−^^3^
Hall symbol: -P 1	Melting point = 461–462 K
*a* = 7.3725 (2) Å	Mo *K*α radiation, λ = 0.71073 Å
*b* = 10.0967 (3) Å	Cell parameters from 9961 reflections
*c* = 20.8173 (7) Å	θ = 2.6–28.3°
α = 98.086 (1)°	µ = 0.42 mm^−^^1^
β = 99.547 (2)°	*T* = 173 K
γ = 106.547 (1)°	Block, colourless
*V* = 1435.62 (8) Å^3^	0.41 × 0.29 × 0.23 mm

### Data collection

**Table:**

Bruker SMART APEXII CCD diffractometer	7109 independent reflections
Radiation source: rotating anode	5967 reflections with *I* > 2σ(*I*)
Graphite multilayer monochromator	*R*_int_ = 0.028
Detector resolution: 10.0 pixels mm^-1^	θ_max_ = 28.3°, θ_min_ = 1.0°
φ and ω scans	*h* = −9→9
Absorption correction: multi-scan (*SADABS*; Bruker, 2009)	*k* = −13→13
*T*_min_ = 0.670, *T*_max_ = 0.746	*l* = −27→27
26328 measured reflections	

### Refinement

**Table:**

Refinement on *F*^2^	Primary atom site location: structure-invariant direct methods
Least-squares matrix: full	Secondary atom site location: difference Fourier map
*R*[*F*^2^ > 2σ(*F*^2^)] = 0.036	Hydrogen site location: difference Fourier map
*wR*(*F*^2^) = 0.098	H-atom parameters constrained
*S* = 1.04	*w* = 1/[σ^2^(*F*_o_^2^) + (0.0476*P*)^2^ + 0.6008*P*] where *P* = (*F*_o_^2^ + 2*F*_c_^2^)/3
7109 reflections	(Δ/σ)_max_ = 0.001
383 parameters	Δρ_max_ = 0.38 e Å^−^^3^
0 restraints	Δρ_min_ = −0.40 e Å^−^^3^

### Special details

**Table:**

Geometry. All esds (except the esd in the dihedral angle between two l.s. planes) are estimated using the full covariance matrix. The cell esds are taken into account individually in the estimation of esds in distances, angles and torsion angles; correlations between esds in cell parameters are only used when they are defined by crystal symmetry. An approximate (isotropic) treatment of cell esds is used for estimating esds involving l.s. planes.
Refinement. Refinement of F^2^ against ALL reflections. The weighted R-factor wR and goodness of fit S are based on F^2^, conventional R-factors R are based on F, with F set to zero for negative F^2^. The threshold expression of F^2^ > 2sigma(F^2^) is used only for calculating R-factors(gt) etc. and is not relevant to the choice of reflections for refinement. R-factors based on F^2^ are statistically about twice as large as those based on F, and R- factors based on ALL data will be even larger.

### Fractional atomic coordinates and isotropic or equivalent isotropic displacement parameters (Å^2^)

**Table:**

	*x*	*y*	*z*	*U*_iso_*/*U*_eq_	
Cl1	0.24968 (8)	0.40295 (6)	0.68981 (2)	0.04342 (13)	
S1	−0.03637 (6)	0.18272 (4)	0.393226 (19)	0.02505 (10)	
O1	0.29244 (17)	0.57988 (12)	0.43797 (6)	0.0290 (2)	
O2	−0.13514 (17)	0.13204 (12)	0.44305 (6)	0.0310 (3)	
O3	−0.14612 (18)	0.18003 (13)	0.32903 (6)	0.0347 (3)	
C1	0.1097 (2)	0.35510 (16)	0.42756 (7)	0.0238 (3)	
C2	0.1844 (2)	0.41316 (16)	0.49781 (7)	0.0228 (3)	
C3	0.1677 (2)	0.36384 (17)	0.55651 (8)	0.0261 (3)	
H3	0.0931	0.2699	0.5563	0.031*	
C4	0.2657 (2)	0.45931 (19)	0.61515 (8)	0.0296 (4)	
C5	0.3744 (2)	0.59807 (19)	0.61716 (8)	0.0328 (4)	
H5	0.4377	0.6592	0.6588	0.039*	
C6	0.3910 (2)	0.64748 (18)	0.55939 (9)	0.0313 (4)	
H6	0.4639	0.7419	0.5597	0.038*	
C7	0.2953 (2)	0.55174 (17)	0.50081 (8)	0.0254 (3)	
C8	0.1795 (2)	0.45846 (17)	0.39433 (8)	0.0270 (3)	
C9	0.1631 (3)	0.4659 (2)	0.32330 (9)	0.0363 (4)	
H9A	0.2897	0.4773	0.3119	0.054*	
H9B	0.1203	0.5465	0.3149	0.054*	
H9C	0.0686	0.3788	0.2960	0.054*	
C10	0.1255 (2)	0.08739 (16)	0.38118 (8)	0.0253 (3)	
C11	0.1953 (3)	0.08322 (18)	0.32311 (8)	0.0312 (4)	
H11	0.1560	0.1310	0.2899	0.037*	
C12	0.3231 (3)	0.00823 (19)	0.31462 (9)	0.0345 (4)	
H12	0.3732	0.0064	0.2754	0.041*	
C13	0.3796 (2)	−0.06433 (18)	0.36201 (9)	0.0327 (4)	
C14	0.3082 (3)	−0.05848 (19)	0.41956 (9)	0.0343 (4)	
H14	0.3458	−0.1076	0.4525	0.041*	
C15	0.1828 (2)	0.01806 (18)	0.42974 (8)	0.0295 (3)	
H15	0.1366	0.0229	0.4697	0.035*	
S2	0.09432 (6)	0.27584 (4)	0.103954 (18)	0.02438 (9)	
Cl2	0.01357 (7)	0.22781 (5)	−0.19394 (2)	0.03793 (11)	
O4	0.36056 (16)	0.63145 (12)	0.05479 (5)	0.0276 (2)	
O6	−0.06739 (16)	0.18020 (12)	0.05491 (6)	0.0302 (3)	
O5	0.06614 (18)	0.33050 (13)	0.16777 (6)	0.0330 (3)	
C17	0.1952 (2)	0.41650 (16)	0.06766 (7)	0.0237 (3)	
C18	0.1834 (2)	0.41055 (16)	−0.00264 (7)	0.0225 (3)	
C19	0.0958 (2)	0.30830 (17)	−0.06083 (8)	0.0259 (3)	
H19	0.0237	0.2146	−0.0597	0.031*	
C20	0.1199 (2)	0.35085 (18)	−0.11983 (8)	0.0271 (3)	
C21	0.2233 (2)	0.48782 (19)	−0.12355 (8)	0.0302 (4)	
H21	0.2341	0.5115	−0.1656	0.036*	
C22	0.3098 (2)	0.58913 (18)	−0.06623 (8)	0.0292 (3)	
H22	0.3808	0.6831	−0.0674	0.035*	
C23	0.2875 (2)	0.54624 (16)	−0.00748 (8)	0.0246 (3)	
C24	0.3028 (2)	0.54992 (17)	0.09942 (8)	0.0260 (3)	
C25	0.3701 (3)	0.62270 (19)	0.16990 (8)	0.0349 (4)	
H25A	0.3051	0.5622	0.1977	0.052*	
H25B	0.3391	0.7113	0.1754	0.052*	
H25C	0.5105	0.6429	0.1834	0.052*	
C26	0.2726 (2)	0.19238 (16)	0.11785 (8)	0.0245 (3)	
C27	0.2712 (2)	0.08041 (17)	0.07094 (8)	0.0298 (3)	
H27	0.1760	0.0494	0.0306	0.036*	
C28	0.4102 (3)	0.01391 (18)	0.08349 (9)	0.0345 (4)	
H28	0.4080	−0.0643	0.0518	0.041*	
C29	0.5527 (2)	0.06008 (18)	0.14183 (9)	0.0326 (4)	
C30	0.5542 (3)	0.1749 (2)	0.18749 (9)	0.0342 (4)	
H30	0.6524	0.2085	0.2271	0.041*	
C31	0.4152 (3)	0.24113 (18)	0.17622 (8)	0.0302 (4)	
H31	0.4168	0.3191	0.2080	0.036*	
C32	0.7015 (3)	−0.0134 (2)	0.15597 (11)	0.0444 (5)	
H32A	0.6896	−0.0838	0.1166	0.067*	
H32B	0.6805	−0.0601	0.1935	0.067*	
H32C	0.8315	0.0559	0.1670	0.067*	
C16	0.5134 (3)	−0.1492 (2)	0.35059 (12)	0.0447 (5)	
H16A	0.4374	−0.2440	0.3255	0.067*	
H16B	0.5848	−0.1559	0.3935	0.067*	
H16C	0.6053	−0.1029	0.3253	0.067*	

### Atomic displacement parameters (Å^2^)

**Table:**

	*U*^11^	*U*^22^	*U*^33^	*U*^12^	*U*^13^	*U*^23^
Cl1	0.0559 (3)	0.0585 (3)	0.0223 (2)	0.0277 (2)	0.00813 (19)	0.00901 (19)
S1	0.02324 (19)	0.02530 (19)	0.02250 (18)	0.00409 (15)	0.00130 (14)	0.00326 (14)
O1	0.0304 (6)	0.0251 (6)	0.0312 (6)	0.0062 (5)	0.0083 (5)	0.0081 (5)
O2	0.0275 (6)	0.0301 (6)	0.0333 (6)	0.0037 (5)	0.0096 (5)	0.0076 (5)
O3	0.0322 (6)	0.0381 (7)	0.0267 (6)	0.0082 (5)	−0.0054 (5)	0.0031 (5)
C1	0.0226 (7)	0.0242 (7)	0.0231 (7)	0.0066 (6)	0.0031 (6)	0.0034 (6)
C2	0.0203 (7)	0.0253 (7)	0.0232 (7)	0.0085 (6)	0.0044 (6)	0.0035 (6)
C3	0.0266 (8)	0.0279 (8)	0.0245 (7)	0.0097 (6)	0.0053 (6)	0.0055 (6)
C4	0.0309 (8)	0.0401 (9)	0.0215 (7)	0.0180 (7)	0.0041 (6)	0.0053 (7)
C5	0.0288 (8)	0.0354 (9)	0.0291 (8)	0.0125 (7)	−0.0018 (7)	−0.0049 (7)
C6	0.0255 (8)	0.0256 (8)	0.0376 (9)	0.0066 (7)	0.0016 (7)	−0.0017 (7)
C7	0.0220 (7)	0.0264 (8)	0.0288 (8)	0.0085 (6)	0.0064 (6)	0.0054 (6)
C8	0.0266 (8)	0.0279 (8)	0.0270 (8)	0.0093 (6)	0.0052 (6)	0.0066 (6)
C9	0.0424 (10)	0.0425 (10)	0.0281 (8)	0.0138 (8)	0.0106 (7)	0.0159 (8)
C10	0.0261 (8)	0.0208 (7)	0.0244 (7)	0.0029 (6)	0.0032 (6)	0.0017 (6)
C11	0.0367 (9)	0.0297 (8)	0.0230 (7)	0.0062 (7)	0.0047 (7)	0.0026 (6)
C12	0.0380 (10)	0.0311 (9)	0.0295 (8)	0.0049 (7)	0.0113 (7)	−0.0017 (7)
C13	0.0294 (9)	0.0213 (8)	0.0408 (9)	0.0018 (6)	0.0071 (7)	−0.0018 (7)
C14	0.0361 (9)	0.0288 (9)	0.0386 (9)	0.0098 (7)	0.0074 (7)	0.0106 (7)
C15	0.0328 (9)	0.0277 (8)	0.0273 (8)	0.0065 (7)	0.0085 (7)	0.0079 (6)
S2	0.02491 (19)	0.02583 (19)	0.02175 (18)	0.00625 (15)	0.00545 (14)	0.00582 (14)
Cl2	0.0461 (3)	0.0422 (3)	0.02349 (19)	0.0162 (2)	0.00331 (17)	0.00018 (17)
O4	0.0278 (6)	0.0234 (6)	0.0282 (6)	0.0048 (5)	0.0039 (5)	0.0039 (4)
O6	0.0248 (6)	0.0302 (6)	0.0310 (6)	0.0032 (5)	0.0029 (5)	0.0065 (5)
O5	0.0377 (7)	0.0378 (7)	0.0258 (6)	0.0121 (5)	0.0126 (5)	0.0070 (5)
C17	0.0243 (7)	0.0247 (7)	0.0223 (7)	0.0080 (6)	0.0047 (6)	0.0053 (6)
C18	0.0206 (7)	0.0249 (7)	0.0234 (7)	0.0086 (6)	0.0046 (6)	0.0066 (6)
C19	0.0257 (8)	0.0252 (8)	0.0255 (7)	0.0075 (6)	0.0040 (6)	0.0045 (6)
C20	0.0276 (8)	0.0325 (8)	0.0226 (7)	0.0130 (7)	0.0047 (6)	0.0035 (6)
C21	0.0323 (9)	0.0376 (9)	0.0278 (8)	0.0159 (7)	0.0117 (7)	0.0134 (7)
C22	0.0284 (8)	0.0276 (8)	0.0347 (9)	0.0092 (7)	0.0105 (7)	0.0113 (7)
C23	0.0223 (7)	0.0252 (8)	0.0269 (7)	0.0085 (6)	0.0052 (6)	0.0051 (6)
C24	0.0251 (8)	0.0264 (8)	0.0269 (8)	0.0090 (6)	0.0051 (6)	0.0054 (6)
C25	0.0369 (10)	0.0325 (9)	0.0279 (8)	0.0065 (7)	0.0013 (7)	−0.0017 (7)
C26	0.0260 (8)	0.0232 (7)	0.0238 (7)	0.0056 (6)	0.0048 (6)	0.0086 (6)
C27	0.0290 (8)	0.0257 (8)	0.0291 (8)	0.0045 (7)	0.0012 (6)	0.0019 (6)
C28	0.0351 (9)	0.0254 (8)	0.0414 (10)	0.0087 (7)	0.0075 (8)	0.0039 (7)
C29	0.0297 (9)	0.0289 (8)	0.0423 (10)	0.0082 (7)	0.0093 (7)	0.0179 (7)
C30	0.0330 (9)	0.0393 (10)	0.0280 (8)	0.0092 (8)	−0.0001 (7)	0.0119 (7)
C31	0.0351 (9)	0.0309 (8)	0.0222 (7)	0.0087 (7)	0.0025 (6)	0.0056 (6)
C32	0.0368 (10)	0.0415 (11)	0.0621 (13)	0.0171 (9)	0.0106 (9)	0.0243 (10)
C16	0.0392 (11)	0.0286 (9)	0.0641 (13)	0.0088 (8)	0.0160 (9)	0.0002 (9)

### Geometric parameters (Å, º)

**Table:**

Cl1—C4	1.7392 (17)	S2—C26	1.7591 (16)
S1—O2	1.4336 (12)	Cl2—C20	1.7443 (16)
S1—O3	1.4353 (12)	O4—C24	1.367 (2)
S1—C1	1.7397 (16)	O4—C23	1.3789 (18)
S1—C10	1.7607 (16)	C17—C24	1.359 (2)
O1—C8	1.370 (2)	C17—C18	1.443 (2)
O1—C7	1.3751 (19)	C18—C23	1.394 (2)
C1—C8	1.359 (2)	C18—C19	1.400 (2)
C1—C2	1.447 (2)	C19—C20	1.379 (2)
C2—C7	1.392 (2)	C19—H19	0.9500
C2—C3	1.394 (2)	C20—C21	1.396 (2)
C3—C4	1.386 (2)	C21—C22	1.382 (2)
C3—H3	0.9500	C21—H21	0.9500
C4—C5	1.393 (3)	C22—C23	1.373 (2)
C5—C6	1.376 (3)	C22—H22	0.9500
C5—H5	0.9500	C24—C25	1.479 (2)
C6—C7	1.383 (2)	C25—H25A	0.9800
C6—H6	0.9500	C25—H25B	0.9800
C8—C9	1.478 (2)	C25—H25C	0.9800
C9—H9A	0.9800	C26—C27	1.382 (2)
C9—H9B	0.9800	C26—C31	1.392 (2)
C9—H9C	0.9800	C27—C28	1.386 (2)
C10—C15	1.384 (2)	C27—H27	0.9500
C10—C11	1.390 (2)	C28—C29	1.391 (2)
C11—C12	1.384 (3)	C28—H28	0.9500
C11—H11	0.9500	C29—C30	1.389 (3)
C12—C13	1.387 (3)	C29—C32	1.502 (2)
C12—H12	0.9500	C30—C31	1.381 (2)
C13—C14	1.387 (3)	C30—H30	0.9500
C13—C16	1.505 (3)	C31—H31	0.9500
C14—C15	1.387 (2)	C32—H32A	0.9800
C14—H14	0.9500	C32—H32B	0.9800
C15—H15	0.9500	C32—H32C	0.9800
S2—O6	1.4351 (12)	C16—H16A	0.9800
S2—O5	1.4364 (12)	C16—H16B	0.9800
S2—C17	1.7399 (16)	C16—H16C	0.9800
			
O2—S1—O3	119.75 (8)	C24—O4—C23	106.77 (12)
O2—S1—C1	106.70 (7)	C24—C17—C18	107.37 (14)
O3—S1—C1	108.68 (8)	C24—C17—S2	127.08 (12)
O2—S1—C10	107.90 (8)	C18—C17—S2	125.53 (12)
O3—S1—C10	107.69 (7)	C23—C18—C19	119.07 (14)
C1—S1—C10	105.23 (7)	C23—C18—C17	104.74 (13)
C8—O1—C7	106.81 (12)	C19—C18—C17	136.18 (15)
C8—C1—C2	107.30 (14)	C20—C19—C18	116.46 (15)
C8—C1—S1	127.15 (12)	C20—C19—H19	121.8
C2—C1—S1	125.53 (12)	C18—C19—H19	121.8
C7—C2—C3	119.56 (14)	C19—C20—C21	123.49 (15)
C7—C2—C1	104.64 (14)	C19—C20—Cl2	118.42 (13)
C3—C2—C1	135.79 (15)	C21—C20—Cl2	118.08 (13)
C4—C3—C2	116.34 (15)	C22—C21—C20	120.25 (15)
C4—C3—H3	121.8	C22—C21—H21	119.9
C2—C3—H3	121.8	C20—C21—H21	119.9
C3—C4—C5	123.26 (16)	C23—C22—C21	116.20 (16)
C3—C4—Cl1	118.16 (14)	C23—C22—H22	121.9
C5—C4—Cl1	118.58 (13)	C21—C22—H22	121.9
C6—C5—C4	120.66 (15)	C22—C23—O4	125.08 (15)
C6—C5—H5	119.7	C22—C23—C18	124.52 (15)
C4—C5—H5	119.7	O4—C23—C18	110.39 (13)
C5—C6—C7	116.12 (16)	C17—C24—O4	110.74 (14)
C5—C6—H6	121.9	C17—C24—C25	134.29 (16)
C7—C6—H6	121.9	O4—C24—C25	114.97 (14)
O1—C7—C6	125.33 (15)	C24—C25—H25A	109.5
O1—C7—C2	110.61 (13)	C24—C25—H25B	109.5
C6—C7—C2	124.05 (16)	H25A—C25—H25B	109.5
C1—C8—O1	110.64 (14)	C24—C25—H25C	109.5
C1—C8—C9	134.21 (16)	H25A—C25—H25C	109.5
O1—C8—C9	115.13 (15)	H25B—C25—H25C	109.5
C8—C9—H9A	109.5	C27—C26—C31	120.64 (15)
C8—C9—H9B	109.5	C27—C26—S2	120.14 (12)
H9A—C9—H9B	109.5	C31—C26—S2	119.21 (12)
C8—C9—H9C	109.5	C26—C27—C28	119.28 (15)
H9A—C9—H9C	109.5	C26—C27—H27	120.4
H9B—C9—H9C	109.5	C28—C27—H27	120.4
C15—C10—C11	120.71 (16)	C27—C28—C29	120.96 (16)
C15—C10—S1	119.69 (13)	C27—C28—H28	119.5
C11—C10—S1	119.59 (13)	C29—C28—H28	119.5
C12—C11—C10	118.78 (16)	C30—C29—C28	118.75 (16)
C12—C11—H11	120.6	C30—C29—C32	120.25 (17)
C10—C11—H11	120.6	C28—C29—C32	121.00 (17)
C11—C12—C13	121.53 (16)	C31—C30—C29	121.04 (16)
C11—C12—H12	119.2	C31—C30—H30	119.5
C13—C12—H12	119.2	C29—C30—H30	119.5
C14—C13—C12	118.62 (16)	C30—C31—C26	119.30 (16)
C14—C13—C16	120.84 (18)	C30—C31—H31	120.3
C12—C13—C16	120.53 (17)	C26—C31—H31	120.4
C13—C14—C15	120.90 (17)	C29—C32—H32A	109.5
C13—C14—H14	119.5	C29—C32—H32B	109.5
C15—C14—H14	119.5	H32A—C32—H32B	109.5
C10—C15—C14	119.43 (16)	C29—C32—H32C	109.5
C10—C15—H15	120.3	H32A—C32—H32C	109.5
C14—C15—H15	120.3	H32B—C32—H32C	109.5
O6—S2—O5	119.79 (7)	C13—C16—H16A	109.5
O6—S2—C17	106.81 (7)	C13—C16—H16B	109.5
O5—S2—C17	108.76 (8)	H16A—C16—H16B	109.5
O6—S2—C26	107.88 (7)	C13—C16—H16C	109.5
O5—S2—C26	107.38 (7)	H16A—C16—H16C	109.5
C17—S2—C26	105.33 (7)	H16B—C16—H16C	109.5
			
O2—S1—C1—C8	−157.13 (14)	O6—S2—C17—C24	155.68 (14)
O3—S1—C1—C8	−26.72 (17)	O5—S2—C17—C24	25.08 (17)
C10—S1—C1—C8	88.40 (16)	C26—S2—C17—C24	−89.78 (15)
O2—S1—C1—C2	25.03 (15)	O6—S2—C17—C18	−26.17 (15)
O3—S1—C1—C2	155.44 (13)	O5—S2—C17—C18	−156.77 (13)
C10—S1—C1—C2	−89.44 (14)	C26—S2—C17—C18	88.37 (14)
C8—C1—C2—C7	0.54 (17)	C24—C17—C18—C23	−0.43 (17)
S1—C1—C2—C7	178.73 (12)	S2—C17—C18—C23	−178.88 (11)
C8—C1—C2—C3	179.77 (17)	C24—C17—C18—C19	−179.32 (17)
S1—C1—C2—C3	−2.0 (3)	S2—C17—C18—C19	2.2 (3)
C7—C2—C3—C4	−0.1 (2)	C23—C18—C19—C20	−0.1 (2)
C1—C2—C3—C4	−179.20 (16)	C17—C18—C19—C20	178.70 (16)
C2—C3—C4—C5	0.7 (2)	C18—C19—C20—C21	−0.6 (2)
C2—C3—C4—Cl1	−179.93 (11)	C18—C19—C20—Cl2	−179.89 (11)
C3—C4—C5—C6	−0.6 (3)	C19—C20—C21—C22	0.6 (2)
Cl1—C4—C5—C6	−179.89 (13)	Cl2—C20—C21—C22	179.89 (13)
C4—C5—C6—C7	−0.3 (2)	C20—C21—C22—C23	0.1 (2)
C8—O1—C7—C6	−178.94 (15)	C21—C22—C23—O4	−179.20 (14)
C8—O1—C7—C2	−0.19 (17)	C21—C22—C23—C18	−0.8 (2)
C5—C6—C7—O1	179.58 (14)	C24—O4—C23—C22	178.55 (15)
C5—C6—C7—C2	1.0 (2)	C24—O4—C23—C18	−0.02 (16)
C3—C2—C7—O1	−179.60 (13)	C19—C18—C23—C22	0.8 (2)
C1—C2—C7—O1	−0.21 (16)	C17—C18—C23—C22	−178.31 (15)
C3—C2—C7—C6	−0.8 (2)	C19—C18—C23—O4	179.40 (13)
C1—C2—C7—C6	178.56 (15)	C17—C18—C23—O4	0.28 (16)
C2—C1—C8—O1	−0.68 (18)	C18—C17—C24—O4	0.44 (17)
S1—C1—C8—O1	−178.84 (11)	S2—C17—C24—O4	178.86 (11)
C2—C1—C8—C9	177.70 (18)	C18—C17—C24—C25	−178.78 (17)
S1—C1—C8—C9	−0.5 (3)	S2—C17—C24—C25	−0.4 (3)
C7—O1—C8—C1	0.55 (17)	C23—O4—C24—C17	−0.27 (17)
C7—O1—C8—C9	−178.17 (14)	C23—O4—C24—C25	179.12 (13)
O2—S1—C10—C15	−19.93 (15)	O6—S2—C26—C27	20.41 (16)
O3—S1—C10—C15	−150.50 (13)	O5—S2—C26—C27	150.81 (14)
C1—S1—C10—C15	93.70 (14)	C17—S2—C26—C27	−93.39 (15)
O2—S1—C10—C11	160.40 (13)	O6—S2—C26—C31	−160.06 (13)
O3—S1—C10—C11	29.84 (15)	O5—S2—C26—C31	−29.66 (15)
C1—S1—C10—C11	−85.96 (14)	C17—S2—C26—C31	86.14 (14)
C15—C10—C11—C12	0.1 (2)	C31—C26—C27—C28	2.0 (3)
S1—C10—C11—C12	179.74 (13)	S2—C26—C27—C28	−178.50 (13)
C10—C11—C12—C13	1.2 (3)	C26—C27—C28—C29	−1.3 (3)
C11—C12—C13—C14	−1.3 (3)	C27—C28—C29—C30	−0.3 (3)
C11—C12—C13—C16	177.91 (17)	C27—C28—C29—C32	179.02 (17)
C12—C13—C14—C15	0.1 (3)	C28—C29—C30—C31	1.2 (3)
C16—C13—C14—C15	−179.11 (17)	C32—C29—C30—C31	−178.07 (17)
C11—C10—C15—C14	−1.2 (2)	C29—C30—C31—C26	−0.6 (3)
S1—C10—C15—C14	179.10 (13)	C27—C26—C31—C30	−1.1 (3)
C13—C14—C15—C10	1.2 (3)	S2—C26—C31—C30	179.42 (13)

### Hydrogen-bond geometry (Å, º)

Cg is the centroid of the C26–C31 ring.

**Table:**

*D*—H···*A*	*D*—H	H···*A*	*D*···*A*	*D*—H···*A*
C15—H15···O2^i^	0.95	2.56	3.251 (2)	130
C27—H27···O6^ii^	0.95	2.55	3.248 (2)	131
C30—H30···O3^iii^	0.95	2.48	3.361 (2)	154
C22—H22···*Cg*^iv^	0.95	2.71	3.572 (2)	136

Symmetry codes: (i) −*x*, −*y*, −*z*+1; (ii) −*x*, −*y*, −*z*; (iii) *x*+1, *y*, *z*; (iv) −*x*+1, −*y*+1, −*z*.
